# *Ancylostoma ailuropodae* n. sp. (Nematoda: Ancylostomatidae), a new hookworm parasite isolated from wild giant pandas in Southwest China

**DOI:** 10.1186/s13071-017-2209-2

**Published:** 2017-06-02

**Authors:** Yue Xie, Eric P. Hoberg, Zijiang Yang, Joseph F. Urban, Guangyou Yang

**Affiliations:** 1 Department of Parasitology, College of Veterinary Medicine, Sichuan Agricultural University, Chengdu, 611130 China; 2 United States Department of Agriculture, Agricultural Research Service, Beltsville Human Nutrition Research Center, Diet, Genomics, and Immunology Laboratory, Beltsville, Maryland 20705 USA; 3 United States Department of Agriculture, Agricultural Research Service, Beltsville Agricultural Research Center, Animal Parasitic Disease Laboratory, Beltsville, Maryland 20705 USA; 4 Department of Civil and Environmental Engineering, University of Maryland, College Park, Maryland 20740 USA

**Keywords:** *Ancylostoma ailuropodae* n. sp, *Ailuropoda melanoleuca*, Morphology, Phylogeny, Ancylostomatidae

## Abstract

**Background:**

Hookworms belonging to the genus *Ancylostoma* (Dubini, 1843) cause ancylostomiasis, a disease of considerable concern in humans and domestic and wild animals. Molecular and epidemiological data support evidence for the zoonotic potential among species of *Ancylostoma* where transmission to humans is facilitated by rapid urbanization and increased human-wildlife interactions. It is important to assess and describe these potential zoonotic parasite species in wildlife, especially in hosts that have physiological similarities to humans and share their habitat. Moreover, defining species diversity within parasite groups that can circulate among free-ranging host species and humans also provides a pathway to understanding the distribution of infection and disease. In this study, we describe a previously unrecognized species of hookworm in the genus *Ancylostoma* in the giant panda, including criteria for morphological and molecular characterization.

**Methods:**

The hookworm specimens were obtained from a wild giant panda that died in the Fengtongzai Natural Reserve in Sichuan Province of China in November 2013. They were microscopically examined and then genetically analyzed by sequencing the nuclear internal transcribed spacer (ITS, ITS1-5.8S-ITS2) and mitochondrial cytochrome *c* oxidase subunit 1 (*cox*1) genes in two representative specimens (one female and one male, FTZ1 and FTZ2, respectively).

**Results:**

*Ancylostoma ailuropodae* n. sp. is proposed for these hookworms. Morphologically the hookworm specimens differ from other congeneric species primarily based on the structure of the buccal capsule in males and females, characterized by 2 pairs of ventrolateral and 2 pairs of dorsolateral teeth; males differ in the structure and shape of the copulatory bursa, where the dorsal ray possesses 2 digitations. Pairwise nuclear and mitochondrial DNA comparisons, genetic distance analysis, and phylogenetic data strongly indicate that *A. ailuropodae* from giant pandas is a separate species which shared a most recent common ancestor with *A. ceylanicum* Looss, 1911 in the genus *Ancylostoma* (family Ancylostomatidae).

**Conclusion:**

*Ancylostoma ailuropodae* n. sp. is the fourth species of hookworm described from the Ursidae and the fifteenth species assigned to the genus *Ancylostoma.* A sister-species association with *A. ceylanicum* and phylogenetic distinctiveness from the monophyletic *Uncinaria* Frölich, 1789 among ursids and other carnivorans indicate a history of host colonization in the evolutionary radiation among ancylostomatid hookworms. Further, phylogenetic relationships among bears and a history of ecological and geographical isolation for giant pandas may be consistent with two independent events of host colonization in the diversification of *Ancylostoma* among ursid hosts. A history for host colonization within this assemblage and the relationship for *A. ailuropodae* n. sp. demonstrate the potential of this species as a zoonotic parasite and as a possible threat to human health. The cumulative morphological, molecular and phylogenetic data presented for *A. ailuropodae* n. sp. provides a better understanding of the taxonomy, diagnostics and evolutionary biology of the hookworms.

## Background

Hookworms (Nematoda: Ancylostomatidae) are one of the most common soil-transmitted helminths, causing serious iron-deficiency anemia and protein malnutrition in humans and domestic and wild mammals [1–3]. Both major genera *Ancylostoma* (Dubini, 1843) and *Necator* Stiles, 1903, relegated to two distinct subfamilies, are responsible for morbidity and socioeconomic burdens [4]. Unlike species in the genus *Necator*, most *Ancylostoma* hookworms are considered to be of greater medical and veterinary importance because of distribution, prevalence, and multiple zoonotic species [2]. Currently there are fourteen valid species identified in the genus *Ancylostoma* that are often considered in the context of the range of hosts that are typically infected. For example, the ‘anthrophilic’ form is limited to *Ancylostoma duodenale* (Dubini, 1843) which principally infects humans. ‘Anthropozoonotic’ forms, capable of circulating among free-ranging wild hosts, some domestic hosts and humans include *Ancylostoma caninum* (Ercolani, 1859), *Ancylostoma braziliense* Gomes de Faria, 1910 and *Ancylostoma ceylanicum* Looss, 1911. Other species, including most of the recognized diversity in the genus are considered to be primarily of veterinary importance, including *Ancylostoma tubaeforme* (Zeder, 1800), *Ancylostoma malayanum* (Alessandrini, 1905), *Ancylostoma pluridentatum* (Alessandrini, 1905), *Ancylostoma paraduodenale* Biocca, 1951, *Ancylostoma kusimaense* Nagayosi, 1955, *Ancylostoma buckleyi* Le Roux & Biocca, 1957, *Ancylostoma taxideae* Kalkan & Hansen, 1966, *Ancylostoma genettae* Macchioni, 1995, *Ancylostoma protelesis* Macchioni, 1995, and *Ancylostoma somaliense* Macchioni, 1995 [5, 6]. It is noteworthy that nearly all of these species can also be found in wildlife, such as *A. duodenale* in *Crocuta crocuta* (Erxleben); *A. caninum* and *A. braziliense* in *Acinonyx jubatus* (Schreber) and *Canis mesomelas* Schreber; *A. ceylanicum* in *Canis lupus dingo* Meyer; *A. paraduodenale* in *Leptailurus serval* (Schreber); *A. malayanum* in *Ursus thibetanus* G. Cuvier; *A. pluridentatum* in *Puma concolor coryi* (Bangs); *A. kusimaense* in *Nyctereutes procyonoides viverrinus* Temminck; *A. taxideae* in *Taxidea taxus taxus* (Schreber); *A. genettae* in *Genetta genetta* (Linnaeus); *A. protelesis* in *Proteles cristata* (Sparrman); and *A. somaliense* in *C. mesomelas* [5–12]. Although a diverse assemblage of carnivorans is recognized as hosts for *Ancylostoma*, only one species had been documented or described previously among the Ursidae [7]; species of the distantly related *Uncinaria* Frölich, 1789, are considered typical in ursine hosts [13].

Recent molecular-based genetic and epidemiological investigations have shown that among certain wild or domestic animal-derived species of *Ancylostoma*, *A. ceylanicum* is becoming the second most common hookworm found to infect and complete its life-cycle in humans [12, 14–18]. Similar transmission and cross-infection cases have been reported for other congeneric species, notably *A. caninum* [12, 19, 20] and *A. braziliense* [12]. Such situations highlight the public health significance of hookworm infection and the necessity to assess their prevalence and distribution, and to identify their wildlife hosts. This has become especially important for wildlife hosts that may have recently adapted to the human environment due to rapid urbanization [14, 21] leading to increased interactions with people in conservation centers and zoological gardens constructed for endangered and valuable animals [22]. Regrettably, little attention has been broadly paid to the species of *Ancylostoma* because of a limited understanding of their diversity, abundance and distribution and the difficulty in etiological and epidemiological sampling in the wild [12, 14].

**Fig. 1:**
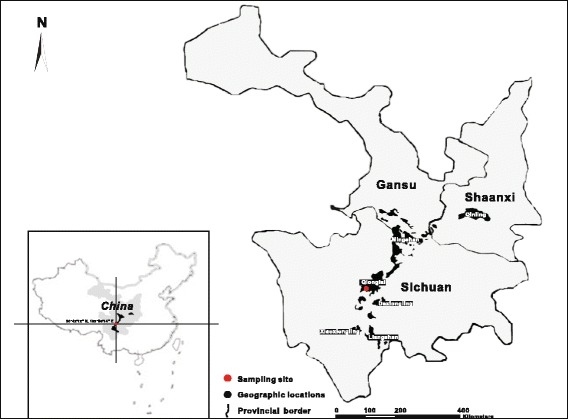
Sampling site in China (*red circle*) for *Ancylostoma ailuropodae* n. sp. in the giant panda. The distribution of the giant panda populations in Shaanxi, Gansu and Sichuan provinces of China is indicated in black with the names of mountain ranges

The giant panda, *Ailuropoda melanoleuca* (David), one of the most endangered and rare species of China, is regarded as one of the preeminent species for wildlife conservation in the world. Higher taxonomic status for these enigmatic carnivorans had been unresolved, until relatively recent decisions that unequivocally placed giant pandas among the Ursidae (e.g. [23–26]). Wild giant pandas currently inhabit six small mountain ranges of China i.e. Qinling, Minshan, Qionglai, Daxiangling, Xiaoxiangling and Liangshan (Fig. 1), with an estimated population size of ~1,864 [27–30]. Since the 1950s, numerous natural reserves, conservation centers, research bases and zoological gardens were specifically established by the Chinese government to protect this threatened species [31]. Some of these wild giant pandas have become closely associated with humans as they are housed for artificial breeding and conservation and biological investigations. Also, some pandas have been displayed publically as the ‘messenger of peace and friendship’ around the world [32]. Although ecological, genetic and etiological studies have shown that the panda faces the threat of extinction due to habitat loss, poor reproduction and low resistance to infectious diseases [33, 34], recent surveys strongly indicate that parasitic infections represent the leading health threat to giant pandas of China [35–40].

Hookworm parasites have been frequently observed in the intestines of wild dead giant pandas since 1995 [28] and the first record, attributed to a species of *Ancylostoma*, was reported by Zhang et al. in 2005 [41]. However, detailed morphological descriptions, determination of taxonomic status and indicators of pathogenicity of the *Ancylostoma* sp. derived from giant panda are lacking. The recent collection of parasites from a wild giant panda that died in the Fengtongzai Natural Reserve in Sichuan Province of China resulted in the recovery of fresh *Ancylostoma* specimens and provided an opportunity to fill some of these gaps in our knowledge. We have used DNA sequence and morphological analysis, applying clear species criteria established in a phylogenetic context [42], to recognize and describe a previously unknown hookworm species from the giant panda. A putative sister-species relationship with the ‘anthropozoonotic’ *A. ceylanicum* suggests a possible zoonotic risk for transmission and infection to humans.

## Methods

### Parasite collection and microscopic examination

In November 2013, a wild female giant panda was found dead in the Fengtongzai Natural Nature Reserve, Sichuan Provence of China (Fig. 1). After a routine necropsy, seventeen hookworm specimens (seven males and ten females) were collected from the small intestine under the Scientific Procedures Premises License for the College of Veterinary Medicine, Sichuan Agricultural University (Sichuan, China). In addition, parasite eggs were isolated from the intestinal content by the centrifuge-flotation method using saturated MgSO_4_ [43]. After washing in physiological saline, the hookworm specimens were either directly fixed in Berland’s fluid (95% glacial acetic acid and 5% formaldehyde) for morphological analysis or stored in 70% ethanol for subsequent molecular profiling. For morphology, the hookworms were identified to the genus level on the basis of the existing taxonomic keys and descriptions of *Ancylostoma* spp. (e.g. [44]). In brief, the worms (*n* = 15; 6 males and 9 females) were prepared as temporary whole mounts in glycerin after clearing in lactophenol and examined under both dissecting and light microscopy at magnifications of 10–40× and 40–200×, respectively; male and female specimens were characterized morphologically including photo-micrographic imaging and morphometrics. Measurements are given in micrometres (μm) unless specified otherwise and presented with the range followed by the mean within parentheses. In addition, some key characteristics of the adults were drawn with the aid of serial photographs for morphological comparison and differentiation from other related species. These specimens including the type-series and vouchers for molecular analyses have been deposited in the Department of Parasitology, Sichuan Agricultural University (accession numbers code GYY-XY).

### Molecular profiles and phylogeny

For molecular analysis, two adult specimens of *Ancylostoma* sp. (one female and one male; sample codes FTZ1 and FTZ2, respectively) preserved in 70% ethanol were air-dried and their mid-body regions (~1 cm) were excised individually for extraction of genomic DNA using the Universal Genomic DNA Extraction Kit (TaKaRa, Dalian, China) according to the manufacturer’s protocol. The cephalic and caudal extremities of each specimen were retained as archived vouchers. The DNA extract was used as template for PCR amplifications at the nuclear internal transcribed spacer ITS1-5.8S-ITS2 region (734 bp) and mitochondrial cytochrome *c* oxidase subunit 1 (*cox*1) locus (393 bp) using primer pairs designed based on the alignments of the relatively conserved regions of the congeneric species *A. ceylanicum*, *A. caninum*, and *A. duodenale* in GenBank. Two PCR primer sets were as follows: ITS1-5.8S-ITS2, forward: 5′-GTC GAA GCC TTA TGG TTC CT-3′ and reverse: 5′-TAA CAG AAA CAC CGT TGT CAT ACT A-3′; *cox*1, forward: 5′-ATT TTA ATT TTG CCT GCT TTT G-3′ and reverse: 5′-ACT AAC AAC ATA ATA GGT ATC ATG TAA-3′. The PCR reactions contained ~20 ng of genomic DNA were performed in 50-μl reaction volumes containing 25 μl 2× Phusion High-Fidelity PCR Master Mix (Finnzymes OY, Espoo, Finland), 3 μl gDNA, 3 μL of each primer and 16 μl of ddH_2_O. PCR cycling conditions carried out in a Mastercycler Gradient 5331 thermocycler (Eppendorf, Germany) were an initial denaturation at 95 °C for 5 min; then for ITS1-5.8S-ITS2, 35 cycles of 95 °C for 30 s, 39.8 °C for 30 s, and 72 °C for 45 s; but for *cox*1, 35 cycles at 95 °C for 30 s, 44.1 °C for 30 s, and 72 °C for 30 s; followed by a final step at 72 °C for 10 min. For each amplification, samples without parasite gDNA and host DNA as negative controls were also included. All PCR products were examined on agarose (1%) gels to verify that they represented the target bands. The corrected gel-isolated amplicons were column-purified and sequenced in both directions using terminator-based cycle sequencing with BigDye chemistry (Applied Biosystems, Foster City, CA, USA) on an ABI 3730 DNA sequencer (Applied Biosystems) in TaKaRa Biotechnology Co. Ltd. (Dalian, China). To ensure maximum accuracy, each amplicon was sequenced three times independently. The consensus sequences were utilized for the following bioinformatic analyses and added to GenBank under the accession numbers KP842923 (FTZ1) and KP842924 (FTZ2) for ITS1-5.8S-ITS2 and KP842921 (FTZ1) and KP842922 (FTZ2) for *cox*1.

Sequences of ITS1-5.8S-ITS2 and *cox*1 of *Ancylostoma* sp. in the present study were separately aligned with reference sequences from closely related species (Table 1), including the congeneric species *A. ceylanicum*, *A. caninum*, *A. duodenale*, *A. braziliense* and *A. tubaeforme* as well as other hookworm species *Necator americanus* (Stiles, 1902), *Uncinaria hamiltoni* Baylis, 1933 [45], *U. lucasi* Stiles & Hassall, 1901, *U. stenocephala* (Railliet, 1884), *U. sanguinis* Marcus, Higgins, Slapeta & Gray, 2014 [46], *Uncinaria* sp., and *Bunostomum phlebotomum* (Railliet, 1900), using the Clustal X 1.83 program [47]. During the procedure, the nucleotide alignment of *cox*1 was further adjusted by a codon-guided protein alignment. Given the presence of the ambiguous regions within these alignments, an online version of GBlocks (http://molevol.cmima.csic.es/castresana/Gblocks_server.html) was also introduced here. After refining the alignments using Gblocks, the sequence datasets were used for phylogenetic analyses using both maximum parsimony (MP) (PAUP* 4.10b [48]) and Bayesian inference (BI) methods (MrBayes 3.2 [49]). In the MP analysis, heuristic searches were executed by branch-swapping utilizing tree-bisection-reconnection (TBR) algorithm and 1,000 random-addition sequence replicates with 10 trees held at each step, and finally the optimal topology with bootstrapping frequencies (BF) was obtained using Kishino-Hasegawa, as described previously [50]. For the BI analysis, the nucleotide substitution model GTR + I + G was determined using the Bayesian Information Criteria (BIC) test in jModeltest v. 2.1.6 [51], and the trees were constructed employing the Markov chain Monte Carlo (MCMC) method (chains = 4) over 100,000 (*cox*1) or 1,000,000 (ITS1-5.8S-ITS2) generations with every 100th (*cox*1) or 1000th (ITS1-5.8S-ITS2) tree being saved; when the average standard deviation of the split frequencies reduced to less than 0.01, 25% of the first saved trees were discarded as “burn-in” and the consensus (50% majority rule) trees were inferred from all remaining trees and further plotted in TreeviewX (http://taxonomy.zoology.gla.ac.uk/rod/treeview.html), with nodal supports expressed as posterior probabilities (PP). The livestock hookworm *B. phlebotomum* was used as outgroup reference and included in each phylogenetic analysis. Paralleled to the phylogenies, among the genus *Ancylostoma* the new hookworm species coupled with *A. ceylanicum*, *A. caninum*, *A. duodenale* and *A. tubaeforme* was also subjected to detection of synonymous and non-synonymous mutations in the mitochondrial *cox*1 gene using their corresponding protein sequences, followed by determination of genetic distances between them using a distance matrix based on the maximum composite likelihood model in MEGA [52].

**Table 1:** Information of *Ancylostoma* species used for molecular identification in the present study

Species	Gender	Host species	Geographical origin^a^		GenBank accession number		Reference
			ITS1-5.8S-ITS2	*cox*1	ITS1-5.8S-ITS2	*cox*1	
*Ancylostoma ailuropodae* n. sp.	Female	Giant pandas	China (Sichuan)	China (Sichuan)	KP842923	KP842921	This study
*A. ailuropodae* n. sp.	Male	Giant pandas	China (Sichuan)	China (Sichuan)	KP842924	KP842922	This study
*Ancylostoma braziliense*	–	Dogs	Brazil (Belo Horizonte)	–	DQ438055	–	e Silva et al. [64]
*A. braziliense*	–	Dogs	Brazil (Belo Horizonte)	–	DQ438056	–	e Silva et al. [64]
*A. braziliense*	–	Dogs	Brazil (Belo Horizonte)	–	DQ438050	–	e Silva et al. [64]
*A. braziliense*	–	Dogs	Brazil (Campo Grande)	–	DQ438060	–	e Silva et al. [64]
*A. braziliense*	–	Dogs	Brazil (Belo Horizonte)	–	DQ438052	–	e Silva et al. [64]
*Ancylostoma caninum*	Male	Humans	–	Japan (Shiga)	–	AB751617	Unpublished
*A. caninum*	Male	Dogs	–	Australia (Townsville)	–	NC_012309	Jex et al. [65]
*A. caninum*	–	Dogs	Brazil (Belo Horizonte)	–	DQ438074	–	e Silva et al. [64]
*A. caninum*	–	Dogs	Brazil (Belo Horizonte)	–	DQ438071	–	e Silva et al. [64]
*A. caninum*	–	Dogs	Brazil (Belo Horizonte)	–	DQ438075	–	e Silva et al. [64]
*A. caninum*	–	Dogs	Brazil (Belo Horizonte)	–	DQ438077	–	e Silva et al. [64]
*A. caninum*	–	Dogs	Brazil (Belo Horizonte)	–	DQ438072	–	e Silva et al. [64]
*Ancylostoma ceylanicum*	Male	Dogs	UK (Nottingham)	–	DQ381541	–	Traub et al. [66]
*A. ceylanicum*	–	Dogs	India (Assam)	–	DQ780009	–	Traub et al. [66]
*A. ceylanicum*	–	Humans	–	Cambodia (Preah Vihear)	–	KF896599	Inpankaew et al. [16]
*A. ceylanicum*	–	Dogs	–	Cambodia (Preah Vihear)	–	KF896602	Inpankaew et al. [16]
*A. ceylanicum*	–	Humans	–	Cambodia (Preah Vihear)	–	KF896604	Inpankaew et al. [16]
*A. ceylanicum*	–	Humans	–	Cambodia (Preah Vihear)	–	KF896601	Inpankaew et al. [16]
*Ancylostoma duodenale*	–	Humans	–	China (Zhejiang)	–	AJ407968	Hu et al. [67]
*A. duodenale*	–	Humans	–	China (Zhejiang)	–	AJ407959	Hu et al. [67]
*A. duodenale*	–	Humans	–	China (Zhejiang)	–	AJ407942	Hu et al. [67]
*A. duodenale*	–	Humans	–	China (Zhejiang)	–	AJ407953	Hu et al. [67]
*A. duodenale*	–	Humans	–	China (Zhejiang)	–	NC_003415	Hu et al. [68]
*A. duodenale*	–	–	–	China (Xiamen)	EU344797	–	Unpublished
*Ancylostoma tubaeforme*	–	Cats	–	Australia (Townsville)	–	AJ407940	Hu et al. [67]
*A. tubaeforme*	–	Cats	–	USA (Michigan)	JQ812691	–	Lucio-Forster et al. [69]
*Uncinaria hamiltoni*	Female	Sea lions	Argentina (Punta Leon)	–	HQ262116	–	Nadler et al. [70]
*U.hamiltoni*	Female	Fur seals	Uruguay (Lobos Island)	–	HQ262109	–	Nadler et al. [70]
*U.hamiltoni*	Female	Fur seals	Uruguay (Cabo Polonio)	–	HQ262100	–	Nadler et al. [70]
*U.hamiltoni*	Female	Sea lions	Uruguay (Cabo Polonio)	–	HQ262119	–	Nadler et al. [70]
*Uncinaria lucasi*	Male	Sea lions	USA (Hazy Island)	–	HQ262131	–	Nadler et al. [70]
*U. lucasi*	Female	Sea lions	Russia (Iony Island)	–	HQ262149	–	Nadler et al. [70]
*U. lucasi*	Female	Sea lions	USA (Hazy Island)	–	HQ262140	–	Nadler et al. [70]
*U. lucasi*	Female	Sea lions	USA (Hazy Island)	–	HQ262138	–	Nadler et al. [70]
*U. lucasi*	Female	Sea lions	USA (Lowry Island)	–	HQ262142	–	Nadler et al. [70]
*U. lucasi*	Female	Fur seals	USA (Reef Rookery)	–	HQ262078	–	Nadler et al. [70]
*U. lucasi*	Male	Fur seals	USA (Adams Cove)	–	HQ262088	–	Nadler et al. [70]
*U. lucasi*	Male	Sea lions	Russia (Iony Island)	–	HQ262154	–	Nadler et al. [70]
*U. lucasi*	Female	Fur seals	Russia (Commander Islands)	–	HQ262067	–	Nadler et al. [70]
*Uncinaria sanguinis*	–	Sea lions	–	Australia (Kangaroo Island)	–	NC_025267	Haynes et al. [71]
*U. sanguinis*	–	Sea lions	–	Australia (Kangaroo Island)	–	KF924756	Haynes et al. [71]
*Uncinaria stenocephala*	Female	Foxes	USA (San Miguel Island)	–	HQ262052	–	Nadler et al. [70]
*U. stenocephala*	Female	Foxes	USA (San Miguel Island)	–	HQ262053	–	Nadler et al. [70]
*U. stenocephala*	Male	Foxes	USA (San Miguel Island)	–	HQ262054	–	Nadler et al. [70]
*U. stenocephala*	Male	Foxes	USA (San Miguel Island)	–	HQ262055	–	Nadler et al. [70]
*Uncinaria* sp.	Female	Elephant seals	Australia (Macquarie Island)	–	HQ262127	–	Nadler et al. [70]
*Uncinaria* sp.	Female	Elephant seals	Australia (Macquarie Island)	–	HQ262130	–	Nadler et al. [70]
*Uncinaria* sp.	Female	Elephant seals	Australia (Macquarie Island)	–	HQ262124	–	Nadler et al. [70]
*Necator americanus*	–	Humans	–	China (Zhejiang)	–	AJ417719	Hu et al. [68]
*N. americanus*	–	Humans	–	China (Zhejiang)	–	NC_003416	Hu et al. [68]
*N. americanus*	–	–	–	Togo (−)	–	AJ556134	Hu et al. [72]
*N. americanus*	–	Humans	–	Central African Republic (−)	AB793527	–	Hasegawa et al. [73]
*N. americanus*	Male	Humans	–	Guatemala (−)	AF217891	–	Nadler et al. [74]
*N. americanus*	–	–	–	China (−)	KM891738	–	Unpublished
*N. americanus*	–	Humans	–	Laos (Thakhek)	LC036565	–	Unpublished
*Bunostomum phlebotomum* (Outgroup)	–	Sheep	China (Heilongjiang)	–	GQ859497	–	Wang et al. [75];
	Male	Calf	–	South Africa (Pretoria)	–	NC_012308	Jex et al. [65]

^a^ Sample localities in parentheses

## Results

**Family Ancylostomatidae Looss, 1905**

**Genus** ***Ancylostoma*** **(Dubini, 1843)**

***Ancylostoma*** ***ailuropodae*** **Yang, Hoberg** **&** **Xie** **n. sp.**

***Type-host***: Giant panda *Ailuropoda melanoleuca* (David) (Mammalia: Carnivora: Ursidae).

***Type-locality***: Fengtongzai Natural Reserve (30°42′12″N, 102°56′14″E), Baoxing, Sichuan Province, China.

***Type-material***: Holotype, adult male (GYY-XY 1301); allotype, adult female (GYY-XY 1308); paratypes, three adult males (GYY-XY 1302-4) and three females (GYY-XY 1309-11). All materials, together with nine vouchers (three males, GYY-XY1305-7; six females, GYY-XY13012-17) containing one male and one female represented by cephalic and caudal extremities, with the mid-body sub-sampled for DNA sequence analysis, are deposited at the Department of Parasitology in Sichuan Agricultural University, Sichuan, China. Collectors: GY Yang, TF Zhang and Y Xie.

***Site in host***: Small intestine (most in the duodenum).

***Representative DNA sequences***: Representative nuclear ribosomal and mitochondrial DNA sequences were deposited in the GenBank database under the accession numbers KP842923–KP842924 (ITS1-5.8S-ITS2) and KP842921–KP842922 (*cox*1).

***ZooBank registration***: To comply with the regulations set out in article 8.5 of the amended 2012 version of the *International Code of Zoological Nomenclature* (ICZN) [53], details of the new species have been submitted to ZooBank. The Life Science Identifier (LSID) of the article is urn:lsid:zoobank.org:pub:A2492E99-AA70-4A58-AB70-7FED78E726A3. The LSID for the new name *Ancylostoma ailuropodae* n. sp. is urn:lsid:zoobank.org:act:2C6B6C1E-5F70-49B7-A303-B4D5AE9C7847.

***Etymology:*** The new species is named for the type-host.

### Description

**Fig. 2:**
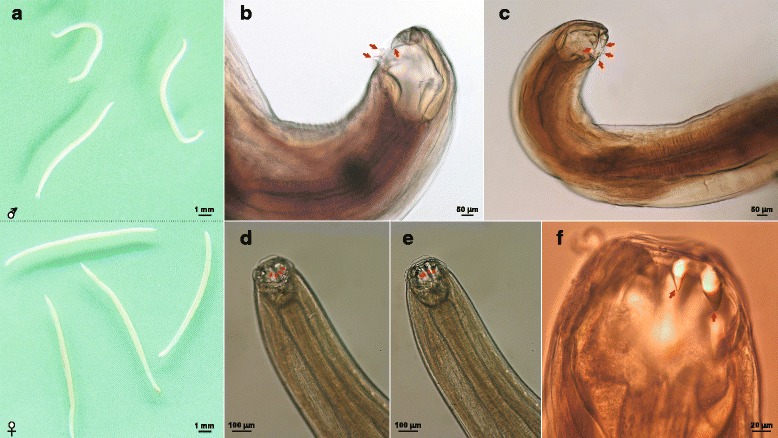
Photomicrographs of adults of *Ancylostoma ailuropodae* n. sp. **a** Total view of males (*top*) and females (*down*); **b**-**f** Cephalic extremity: lateral view of mouth (**b** and **c**), showing dorsolateral and ventrolateral teeth; dorsoventral view of mouth (**d-f**), showing dorsolateral (**d**) and ventrolateral (**e** and **f**) teeth with their positions, shapes and sizes. The arrangements of dorsolateral (2 pairs; **b**-**d**) and ventrolateral (2 pairs; **b**, **c**, **e** and **f**) teeth are indicated by *red* arrows

***General.*** Slender, relatively small nematodes of white coloration in life (Fig. 2a). Body cylindrical, tapering toward cephalic and caudal extremities with fine transversely striated cuticle; head oriented dorsally in males and females. Buccal capsule widening posteriorly to prominent oral aperture, possessing two pairs of ventrolateral teeth and two pairs of triangular dorsolateral teeth (Fig. 2b-f). Ventrolateral teeth vary in size and shape, with small, sub-aduncate inner and large triangular outer teeth extending dorsally. Dorsal gland well developed, associated with rod-like oesophagus, slightly swollen posteriorly, terminating in a lobed valve at junction with intestine (Fig. 4a, b). Nerve-ring at midlevel of oesophagus. Cervical papillae well developed, conical, situated posterior to level of nerve-ring. Excretory pore opens at level between cervical papillae and nerve-ring (Fig. 5a1, 2).

**Fig. 3:**
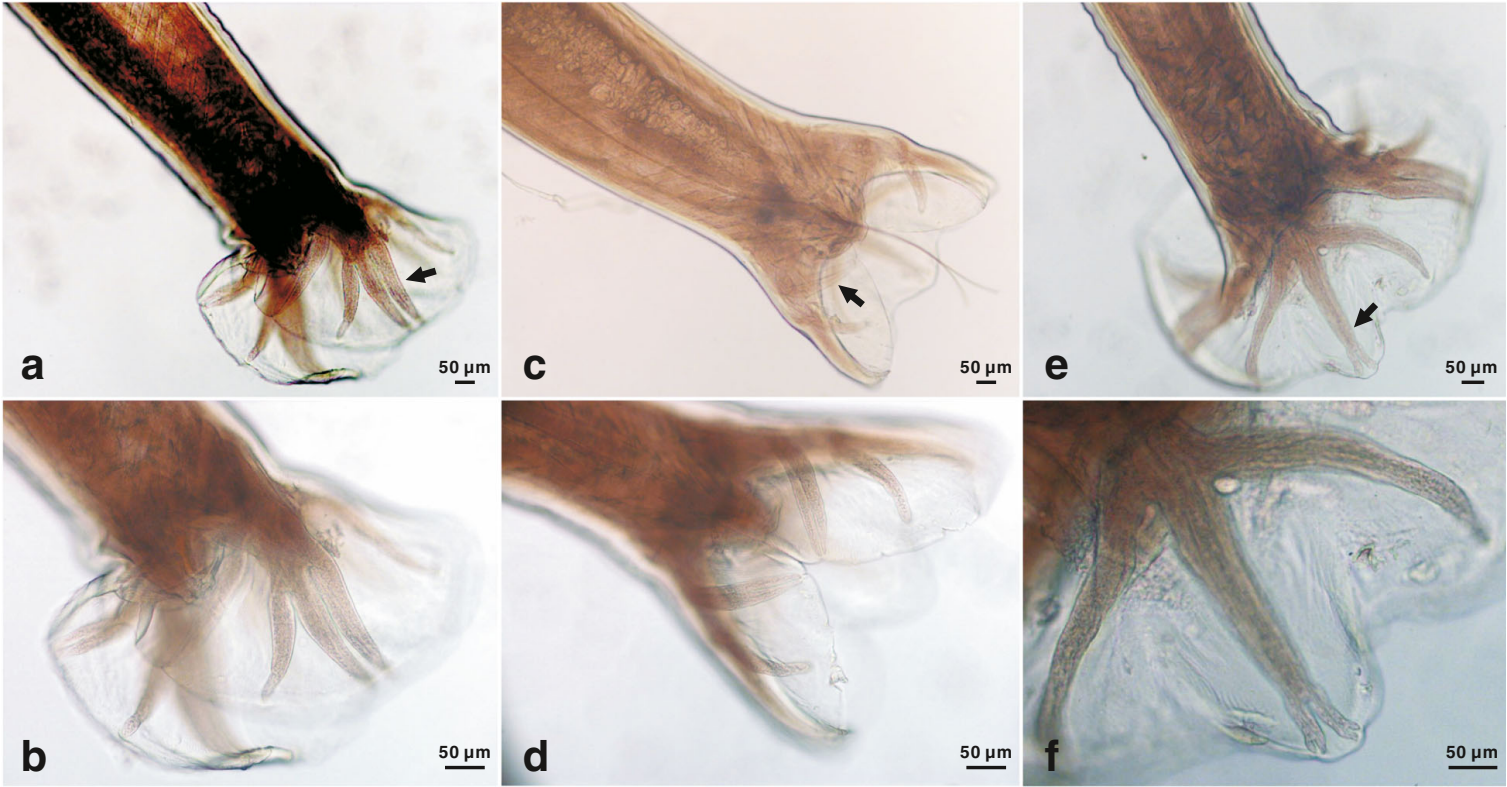
Photomicrographs of *Ancylostoma ailuropodae* n. sp. male, caudal extremity. **a**, **b** Lateral view of bursa showing position of lateral rays and genital cone. **c**, **d** Ventral view of bursa showing configuration of antero- and postero-ventral rays. **e**, **f** Dorsal view of bursa showing relationships of the dorsal and externodorsal rays; note configuration of the bifurcations of the dorsal ray. Arrows in **a**, **c** and **e** denote the rays which are magnified in panels **b**, **d** and **f**, respectively

**Fig. 4:**
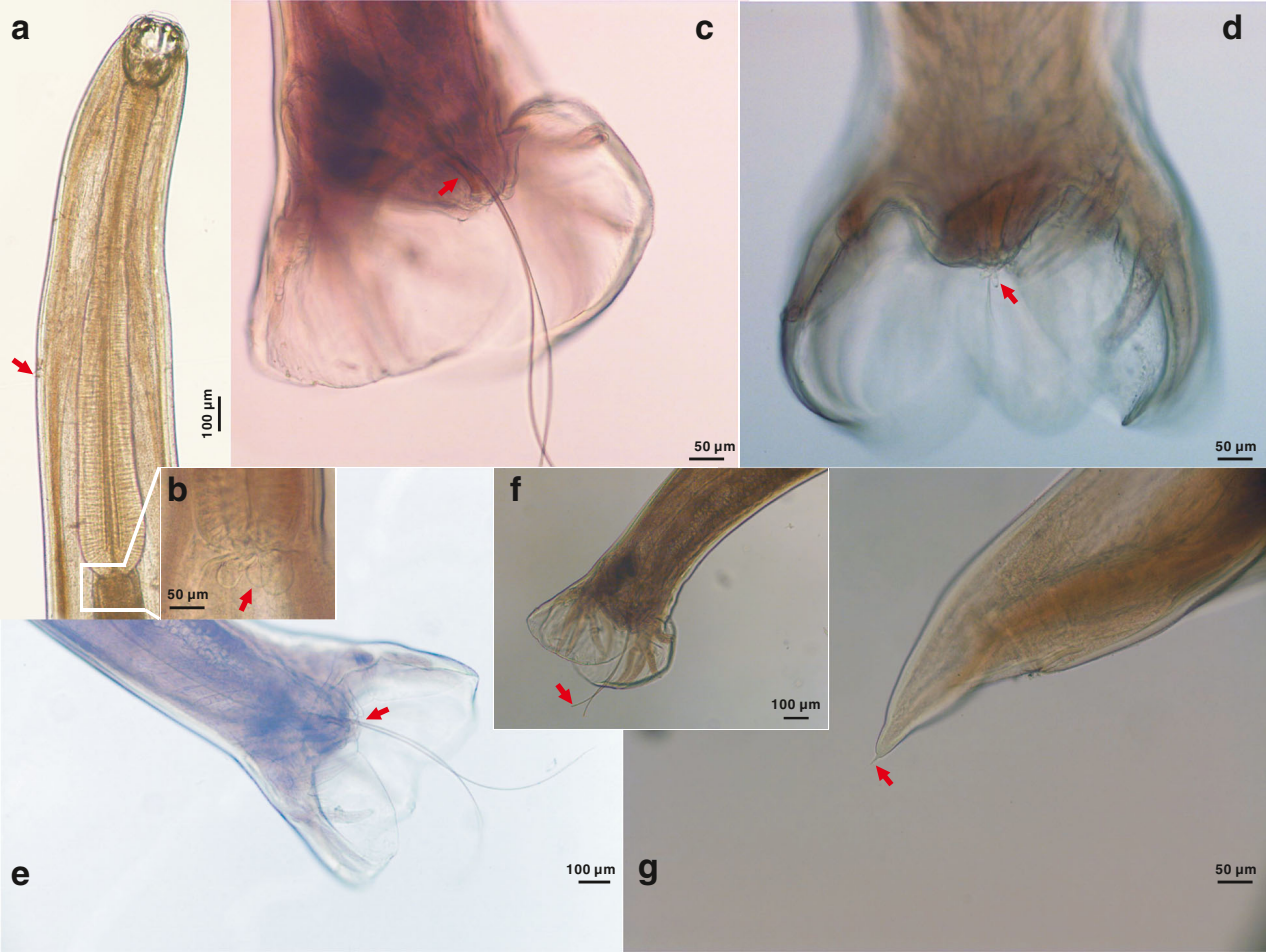
Photomicrographs of adults of *Ancylostoma ailuropodae* n. sp. **a** Dorsoventral view of anterior region of female, showing buccal capsule and entire oesophagus. **b** Lobed valves between oesophagus and intestine. **c** Ventrolateral view of male tail, showing gubernaculum. **d** Ventral view of male tail, showing cloacal papillae. **e**, **f** Ventral and ventrolateral views of male tail, showing spicules from both proximal (**e**) and distal (**f**) extremities. **g** Lateral view of female tail with spine-like point. Arrows indicate some small structures, including cervical papillae (**a**), lobed valve (**b**), gubernaculum (**c**), cloacal papillae (**d**), spicules (**e**, **f**) and spine-like point of female tail (**g**)

**Fig. 5:**
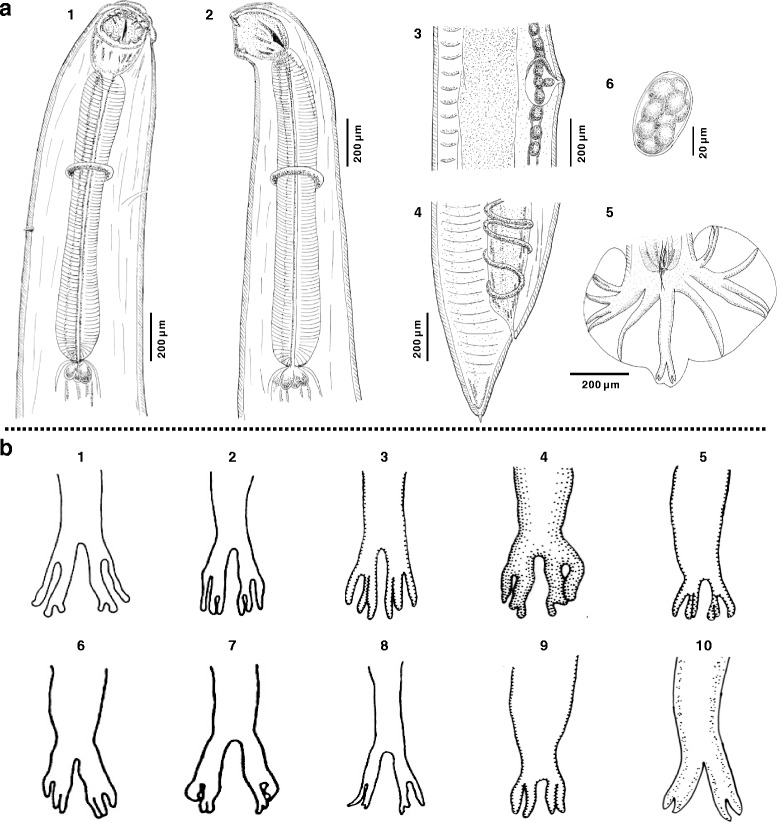
Line drawings of *Ancylostoma ailuropodae* n. sp. and comparison of dorsal rays among *Ancylostoma* spp. **a** Morphological structures of *A. ailuropodae* n. sp.: 1, dorsoventral view of anterior region; 2, lateral view of anterior region; 3, lateral view of female vulval region; 4, lateral view of female caudal region; 5, dorsal view of male caudal region; 6, egg. **b** Ten *Ancylostoma* species for comparison of dorsal rays: 1, *A. taxideae* [10]; 2, *A. duodenale* [9]; 3, *A. paraduodenale* [6]; 4, *A. caninum* [58]; 5, *A. malayanum* [7]; 6, *A. kusimaense* [9]; 7, *A. ceylanicum* [9, 11]; 8, *A. braziliense* [9, 11]; 9, *A. tubaeforme* [58]; 10, *A. ailuropodae* n. sp

***Male.*** [Based on the holotype and three males.] Body length 8.60–12.00 (10.30) mm, maximum width at mid-body 500–520 (510). Buccal capsule 180–220 (200) long, 120–160 (140) wide in dorsoventral view; oesophagus 960–1,500 (1,230) long, 150–190 (170) wide; oesophageal length 12% of total body. Cervical papillae 600–750 (680), excretory pore 500–580 (530), nerve-ring 390–520 (425) posterior to cephalic extremity. Copulatory bursa well developed, broader than long; dorsal lobe small with lateral lobes projecting in direction of lateral trunks (Figs. 3a-f, 5a5). Dorsal ray thick, 280–390 (350) in length, 40–60 (52) in maximum width; bifurcating at 270–295 (280) from anterior into 2 branches; each branch further dividing into 2 sub-branches; externodorsal rays arcuate, arising from dorsal ray at same level (Figs. 3e-f, 5a5). Lateral rays slender, tapering, and arcuate with a common stem. Anterolateral ray bending anteriad, with medio- and posterolateral rays projecting in parallel, extending to edge of bursa (Figs. 3a-b, 5a5). Antero- and posteroventral rays merge at base and then divide, continuing parallel deep into cleft (Figs. 3c-d, 5a5). Spicules tawny colored, paired, equal, filiform, 2,000–2,900 (2,450) long (Figs. 4e-f, 5a5). Gubernaculum fusiform, 80–120 (90) long, 12–20 (16) wide (Figs. 4c, 5a5). Cloacal papillae (*n* = 7) (Figs. 4d, 5a5): 1 pair disposed dorsally, 1 pair laterally, 3 single papillae ventrally.

***Female.*** [Based on the allotype and three females.] Body 9.80–16.00 (12.90) mm long, with maximum width at mid-body 560–740 (650); width at anus 270–340 (285). Buccal capsule 170–250 (210) long, 130–190 (160) wide in dorsoventral view; oesophagus 1,280–1,320 (1,300) long, 170–250 (200) in maximum width near base. Cervical papillae 800–1,230 (900), excretory pore 760–950 (820), nerve-ring 600–650 (620) posterior to cephalic extremity. Vulva opens ventrally in posterior third of body, at 2,450–4,686 (3,480) from caudal extremity; vagina relatively short. Female reproductive system amphidelphic, with poorly differentiated vestibule, paired sphincters and infundibula confluent with uterine and ovarian stems (Fig. 5a3). Tail 90–370 (230) long, terminating in acute, spine-like point 9–25 (17) in length (Figs. 4g, 5a4). Eggs oval, 54–71 × 28–38 (62 × 33) (*n* = 20) (Fig. 5a6).

### Remarks

*Ancylostoma ailuropodae* n. sp. is established based on comparisons to available descriptions among congeners in the global fauna [6, 7, 9, 10, 54–62]. *Ancylostoma ailuropodae* is unequivocally differentiated from congeners by structural characteristics of male and female specimens including body size, arrangement, number and dimensions of buccal teeth and shape of the buccal capsule, and in males by the configuration of the dorsal ray and bursa and lengths of spicules and gubernaculum, respectively (see Table 2 and Fig. 5b). Of note, tooth-number appears to represent one of the key morphological characters separating *A. ailuropodae* from other species of *Ancylostoma*. Specifically, (i) *A. ailuropodae* differs from *A. caninum*, *A. tubaeforme* and *A. taxideae* by the number (2 *vs* 3 pairs) of ventrolateral teeth; and (ii) from *A. ceylanicum*, *A. braziliense*, *A. duodenale*, *A. kusimaense*, *A. paraduodenale* and *A. malayanum* by the number (2 *vs* 0/1 pairs) of triangular dorsolateral teeth. Furthermore, the shape of the dorsal rays appears to be another potential species-specific morphological indicator (Fig. 5b). Specimens of *A. ailuropodae* n. sp. vary from *A. tubaeforme* by differences in cleft length of two digitations in each branch (Fig. 5b9 and 10) and further from *A. taxideae*, *A. duodenale*, *A. paraduodenale*, *A. caninum*, *A. malayanum*, *A. kusimaense*, *A. ceylanicum* and *A. braziliense* by the absence of a third digitation in each branch (Fig. 5b1–8 and 10). Verified specimens of *A. genettae*, *A. protelesis* and *A. somaliense* have not yet been described and these three species were not included in the comparison above. Notably, the adults of both *A. pluridentatum* and *A. buckleyi* can be distinguished from the new species by the number of ventrolateral teeth, given that *A. pluridentatum* has only one pair while *A. buckleyi* has three pairs according to the original descriptions (e.g. [60, 63]). Based on these morphological attributes, *A. ailuropodae* is considered to be a previously unrecognized species within the genus *Ancylostoma*.

**Table 2:** Key comparisons between *A. ailuropodae* n. sp. and other congeneric *Ancylostoma* spp

Species	Body size (mm)	Ventrolateral teeth	Spicules (μm)	Gubernaculum (μm)	Hosts	References
*A. caninum*	M: 11.0–13.0 × 0.34–0.39 (11.7 × 0.37); F: 14.0–20.5 × 0.50–0.56 (17.0 × 0.52)	3 pairs	730–960 (860)	nr	Dogs; cats; humans; wild canids and felids	Burrows [58]
*A. ceylanicum*	M: 7.91 ± 0.04 × 0.35 ± 0.02; F: 9.48 ± 0.81 × 0.42 ± 0.04	2 pairs (outer large; inner very small)	740 ± 20	77 ± 1.64 × 10	Dogs; cats; humans; wild canids and felids	Yoshida [9]
*A. braziliense*	M: 6.84 ± 0.50 × 0.24 ± 0.02; F: 8.67 ± 0.68 × 0.34 ± 0.01	2 pairs(outer large; inner minute)	800 ± 70	73 ± 1.94 × 10 ± 0.44	Humans	Yoshida [9]; Norris[59]
*A. duodenale*	M: 10.67 ± 0.17 × 0.47 ± 0.03; F: 12.67 ± 1.12 × 0.64 ± 0.03	2 pairs (similar in both size and shape)	1,800 ± 90	131 ± 1.49 × 13 ± 0.42	Humans	Yoshida [9]
*A. kusimaense*	M: 7.82 ± 0.20 × 0.28 ± 0.01; F: 9.12 ± 0.55 × 0.33 ± 0.02	2 pairs (outer large; inner small)	840 ± 4	84 ± 0.71 × 10 ± 0.44	Raccoon dogs	Yoshida [9]
*A. tubaeforme*	M: 6.84 ± 0.50 × 0.24 ± 0.02; F: 8.67 ± 0.68 × 0.34 ± 0.01	3 pairs	1,100–1,470 (1,290)	nr	Cats	Burrows [58]
*A. paraduodenale*	M: 5.0–8.0 × 0.21–0.24 (6.8 × 0.23): F: 6.5–8.5 × 0.26–0.32 (7.7 × 0.29)	2 pairs (outer stouter than inner)	1,100–1,500 (1,250)	80 × 20	Servals	Biocca [6]
*A. malayanum*	M: 11.02–13.80 × 0.46–0.51; F: 20.40 × 0.54	2 pairs (outer large, vertical; inner small, subaduncate; one pair of triangular dorsolateral teeth)	2,490–2,620	112^a^	Bears	Wu et al. [7]
*A. taxideae*	M: 8.37 ± 1.92 × 0.32 ± 0.02; F: 16.05 ± 1.30 × 0.46 ± 0.05	3 pairs (one pair of triangular dorsolateral teeth)	1,470 ± 87	138 ± 2 × 15	Badgers	Kalkan & Hansen [10]
*A. ailuropodae* n. sp.	M: 10.30 ± 1.70 × 0.51 ± 0.01; F: 12.90 ± 3.10 × 0.63 ± 0.09	2 pairs (similar in both size and shape; two pairs of triangular dorsolateral teeth)	2,000–2,900 (2,450)	80–120 × 12–20 (90 × 16)	Giant panda	This study

*Abbreviation*: *nr* not reported; the source paper presented no data on the species under consideration

^a^ Only the length of gubernaculum was found in the original description [7]

### Molecular characterization

To further probe the taxonomic position of *A. ailuropodae*, both nuclear ITS1-5.8S-ITS2 and mitochondrial *cox*1 sequences from two representative specimens (codes FTZ1 and FTZ2, respectively) were obtained and subjected to sequence characterization and phylogenetic analyses.

#### Sequence characterization

For ITS1-5.8S-ITS2, the 734 bp sequences from FTZ1 and FTZ2 were identical and had 52.2% A + T content. BLAST analysis revealed that *A. ailuropodae* shared the highest identity with *A. ceylanicum* (99.6%), followed by 98.8% identity with *A. duodenale*, 97.2% with *A. tubaeforme*, 95.8% with *A. caninum*, and 92.6% with *A. braziliense*. Based on the identities, there were a total of 59 variable positions found in the pairwise alignment of ITS1-5.8S-ITS2, including 17 parsimony-informative and 42 singleton sites (data not shown). Within *cox*1 sequences, same base composition (A = 23.4%; C = 9.7%; G = 22.6%; T = 44.3%) and sequence length (393 bp) were also observed in these two representative individuals of *A. ailuropodae*, with an A + T content of 67.7%, a typical mitochondrial nucleotide feature in nematodes (towards AT). BLAST search against GenBank/DDBJ/EMBL databases once again showed the highest nucleotide identity existing between the new species and *A. ceylanicum* (92.6%), followed by 89.2% identity between *A. ailuropodae* and *A. tubaeforme*, 88.6% between *A. ailuropodae* and *A. duodenale*, and 86.0% between *A. ailuropodae* and *A. caninum*, together corresponding to 99.2–100% identities at the amino-acid level. In terms of identity comparisons, there were a total of 78 variable positions in the 378 bp pairwise alignment, including 28 parsimony-informative and 50 singleton sites.

**Fig. 6:**
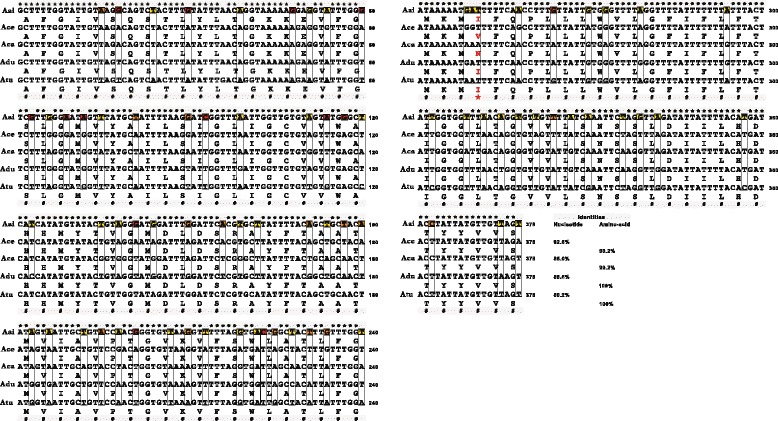
Simultaneous alignments of nucleotide and amino-acid sequences of mitochondrial *cox*1 genes from *Ancylostoma ailuropodae* n. sp. and its congeneric species. For the alignments, the nucleotide sequences of *cox*1 genes were retrieved from the GenBank database (species and accession numbers are indicated in parentheses): Aai (*A. ailuropodae* n. sp.; KP842921), Ace (*A. ceylanicum*; KF896601), Aca (*A. caninum*; AB751617), Adu (*A. duodenale*; NC_003415), and Atu (*A. tubaeforme*; AJ407940). The corresponding protein sequences were deduced based on the Invertebrate Mitochondrial Code. Both nucleotide and amino-acid sequences were aligned with Clustal X 1.83 program. Regions of identity in either nucleotide (*) or amino-acid (#) are indicated. Variable base loci in Aai unique for *A. ailuropodae* n. sp. are highlighted in *red*; those shared between *A. ailuropodae* n. sp. and one of *A. ceylanicum*, *A. duodenale*, *A. caninum* and *A. tubaeforme* are highlighted in *orange*; and those shared between *A. ailuropodae* n. sp. and any two or three of these four congeneric species are highlighted in *yellow*. The non-synonymous substitutions A/G^250^ in *A. ceylanicum* and T/A^251^ in *A. caninum* as well as their amino-acid changes: I (Ilu)/V (Val) and I (Ilu)/N (Asn) are noted in red with a red star. Percentages of nucleotide and amino-acid identities with respect to Aai are shown at the end of each sequence

Further, we located these sites and determined if there were non-synonymous substitutions apparent *via* comparison of their protein sequences, and the results are shown in Fig. 6. Out of 78 variable base sites, 13 were unique for *A. ailuropodae* (in red); 16 were identical between *A. ailuropodae* and one of *A. ceylanicum*, *A. duodenale*, *A. caninum* and *A. tubaeforme* (in orange); and 49 were shared between *A. ailuropodae* and any two or three of these four congeneric species (in yellow). Among the 49 variable sites, however, the non-synonymous substitutions A/G^250^ in *A. ceylanicum* and T/A^251^ in *A. caninum* led to their amino acid changes: I (Ilu) → V (Val) in the former and I (Ilu) → N (Asn) in the latter (see Fig. 6). In addition, analysis of genetic distances using maximum composite likelihood estimates placed *A. ailuropodae* close to *A. ceylanicum* with the minimum interspecific evolutionary divergence (0.084), compared with 0.121 evolutionary divergence to *A. tubaeforme*, 0.127 to *A. duodenale*, and 0.151 to *A. caninum* (not shown).

#### Phylogenetic characterization

**Fig. 7:**
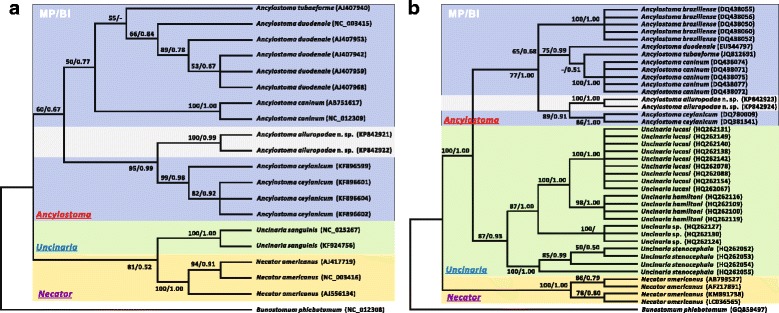
Phylogenetic relationships of hookworms isolated from the giant panda with the related hookworms in the family Ancylostomatidae. Phylogeny was inferred on the basis of mitochondrial *cox*1 (**a**) and nuclear ITS1-5.8S-ITS2 (**b**) sequences using both maximum parsimony (MP) and Bayesian inference (BI) methods. The livestock hookworm *Bunostomum phlebotomum* represented the outgroup species. Taxa belonging to the three major genera including *Ancylostoma*, *Uncinaria* and *Necator* in the family Ancylostomatidae are indicated by differently colored rectangles and shown in both phylogenetic topologies. The numbers along the branches indicate bootstrap values resulting from different analyses in the order MP/BI; values less than 50% are shown as “-”

Phylogenetic relationships between *A. ailuropodae* and other species were inferred from the respective sequences of ITS1-5.8S-ITS2 and *cox*1 using both MP and BI algorithms and their corresponding tree topologies are shown in Fig. 7. Although the two consistent structures (MP/BI) topologically varied from each other due to the different reference species included, both trees provided an identical, robust phylogenetic resolution for *A. ailuropodae* within the genus *Ancylostoma* and for the genus *Ancylostoma* within the family Ancylostomatidae. Specifically, (i) the two *A. ailuropodae* specimens clustered together as a monophyletic group that was separated from the other *Ancylostoma* species. (ii) When the congeneric species *A. ceylanicum*, *A. caninum A. duodenale* and *A. tubaeforme* were considered in our *cox*1-based analysis (Fig. 7a), *A. ailuropodae* and *A. ceylanicum* were more closely related to each other than to *A. caninum*, *A. tubaeforme* and *A. duodenale*, with robust support for tree topology (BP = 95 and PP = 0.99). (iii) When another species, *A. braziliense*, was added to re-construct this phylogenetic relationship using the ITS1-5.8S-ITS2 data (Fig. 7b), *A. ailuropodae* remained as the putative sister of *A. ceylanicum*, regardless of isolate origins (one from the UK and another from India; see Table 1), with high statistical support (BP = 89 and PP = 0.91), which was in agreement with the inferences from the *cox*1 gene analysis (see Fig. 7a). (iv) The inter-relationships of *A. ailuropodae*, *A. ceylanicum*, *A. caninum*, *A. duodenale*, *A. braziliense* and *A. tubaeforme* in the genus *Ancylostoma*; *U. sanguinis*, *U. hamiltoni*, *U. lucasi*, *U. stenocephala* and *Uncinaria* sp. in the genus *Uncinaria*; and *N. americanus* in the genus *Necator*, demonstrated phylogenetic stability of these monophyletic groups, with the current analyses being consistent with previously proposed molecular phylogenies of the hookworms based on the nuclear ribosomal and mitochondrial DNA data [64–75].

## Discussion

Hookworms in the genus *Ancylostoma* cause significant medical and veterinary disease (ancylostomiasis) in various hosts including humans and domestic and wild mammals [2, 71]. Recent epidemiological surveys revealed that some wild animal-derived species of *Ancylostoma* are emerging as important helminthic zoonotic agents because of rapid urbanization and increased human-wildlife interactions [11, 13–21]. The giant panda, for example, is an endangered and rare wild species in China that has been artificially protected and even partially housed for decades due to habitat loss [33]. Clinically unidentified specimens of *Ancylostoma* in giant pandas had been confirmed by veterinarians and wildlife biologists since the last century, but their potential zoonotic importance remains to be defined [41]. In the present study, *A. ailuropodae* n. sp. was isolated from the giant panda, morphologically characterized and demonstrated to be closely related to the anthropozoonotic *A. ceylanicum* by molecular analysis.

In general, morphological identification is a conventional and authoritative approach to define a new nematode parasite species. Concerning the genus *Ancylostoma,* several common species can be morphologically differentiated by key characters such as body size, teeth of the buccal capsule and shape of bursal rays (see Table 2 and Fig. 5b; cf. [9]). Similarly, specimens of *A. ailuropodae* from giant pandas are separated from other hookworms on the basis of either ventrolateral and dorsolateral teeth or dorsal rays, supporting the previous conclusions that teeth and rays were reliable morphological indicators in the differential diagnosis of *Ancylostoma* spp. [55, 56]. Among this assemblage, it is important to note that *A. ailuropodae* is clearly structurally distinct from *A. malayanum*, the only other species of *Ancylostoma* known in ursid hosts (e.g. *Ursus thibetanus*) (Table 2), with the implication that each of these species endemic to China may be more closely related to other congeners within the genus. Specimens upon which the description and differentiation of *A. ailuropodae* n. sp. was based were restricted to fully developed adults and eggs. Further work, using a combined laboratory-egg cultivation and Baermann technique, to describe the morphology of developmentally advanced larval stages is needed to complement morphological characteristics of the new species, and to provide valuable information assisting in species identification and differentiation in this genus [55, 76].

Following our morphological evidence, *A. ailuropodae* from giant pandas was further confirmed as an independent species by molecular analysis. For example, the internal transcribed spacer region (ITS1-5.8S-ITS2) of the nuclear ribosomal DNA is regarded as an appropriate genetic marker to resolve nematode relationships at the species level [77]. Pairwise comparisons of ITS1-5.8S-ITS2 in *A. ailuropodae* with congeneric species available in the GenBank database revealed a species-specific sequence feature (containing 59 variable informative sites) and overall identity of 92.6–99.6% among *A. ceylanicum*, *A. tubaeforme*, *A. caninum* and *A. braziliense*. Furthermore, high bootstrap support was evident, based on phylogenetic analysis of ITS1-5.8S-ITS2 that demonstrated monophyly of *A. ailuropodae* as the putative sister of *A. ceylanicum* (see Fig. 7b).

Critically, similar conclusions were reinforced by analysis of the mitochondrial *cox*1 gene. It should also be noted that *cox*1 analysis was included because recent studies of the substitution patterns for nematode mitochondrial genes (e.g. *cox*1 and *nad*4) revealed that they have utility in identifying and differentiating novel or cryptic species among closely related taxa due to assumed faster evolutionary rates than nuclear genes, features of maternal inheritance and absence of recombination [78–80]. Compared to the nuclear ITS, the *cox*1 of *A. ailuropodae* appeared to have more variable informative sites (*n* = 78, including 13 unique loci). Nevertheless, results based on *cox*1 were consistent with inference from ITS, in revealing a sister-species relationship with *A. ceylanicum* among a broader assemblage of congeners in the genus. Phylogenetic analysis of *cox*1 data (Fig. 7a) also supported the contention that *A. ailuropodae* n. sp. is an independent species which is clearly differentiated from *A. ceylanicum*, *A. caninum*, *A. tubaeforme* and *A. duodenale*.

Based on the results from integrated molecular and morphological comparisons, we propose that *A. ailuropodae* of giant pandas is a previously unrecognized and separate species that is closely related to the anthropozoonotic *A. ceylanicum* within the genus *Ancylostoma*. Additional information regarding the ultrastructure and genomics of this species and other related hookworms is still required. Broader taxonomic comparisons can provide an increasingly precise morphological and molecular basis for species recognition among hookworms. In addition, there were two non-synonymous base substitutions detected in *cox*1 genes of *A. ceylanicum* (A/G^250^) and *A. caninum* (T/A^251^) (Fig. 6) that were confirmed to be fixed and species-specific after homologous comparisons with other *A. ceylanicum* or *A. caninum* isolates from two sites in the same geographic area.

*Ancylostoma ailuropodae* identified here is the fourth hookworm to be described from the Ursidae. Previously, the hookworm *Uncinaria yukonensis* (Wolfgang, 1956) was characterized in black bears and *Uncinaria rauschi* (Olsen, 1968) in grizzly and black bears [81, 82]. On the basis of comparisons of morphometric and distribution data of ursine hookworms as well as the historical biogeography of bears, Catalano et al. [13] proposed that there was a relatively recent host-switching event of *U. rauschi* from black bears to grizzly bears.

The occurrence of *A. ailuropodae* appears consistent with speciation following a host colonization event to giant pandas apparently from a carnivoran source in sympatry, and further indicates a history of independent association with ursine hosts for the broader ancylostomatid hookworm assemblage. The timing and geographic source for these hookworms cannot be elucidated based on the currently available data and the reduced and relictual distribution for pandas, but a history of host colonization is compatible with the current tree topology (for parasites and hosts) and distribution of carnivore hosts for other species of *Ancylostoma* (e.g. [24]). We suggest that acquisition of *Ancylostoma* by giant pandas likely occurred prior to 7 million years ago (MYA) when a shift from an omnivorous diet to one dominated strictly by bamboo (by 2.4 MYA) was underway [25].

Divergence of *A. ailuropodae* appears to have occurred prior to acquisition of *A. ceylanicum* by humans in Southeast Asia, and prior to the intense bottlenecking of giant panda populations that has characterized the past century (cf. [33, 83] for details about the history of giant pandas). This interpretation is significant, as it would relate to the historical independence of *A. ailuropodae* and *A. ceylanicum* before the current intensified conservation campaign for maintaining giant pandas, and the potential for cross-transmission of both hookworm species when infected humans are in contact. The unique niche and specialized bamboo-feeding habits of giant pandas suggest that colonization in ecological time, related to the source or origin of *A. ailuropodae*, was unlikely given relative isolation with respect to a sympatric assemblage of carnivorans or other mammals that may serve as hosts for species of hookworms [25, 26, 33]. Parasitological inventory among potential carnivoran hosts in Sichuan and nearby regions remains necessary to demonstrate that *A. ailuropodae* has a narrow host range and may now be limited to the giant panda [84]; apparent narrow host range, however, does not preclude the potential or capacity for contemporary host switches to humans as a zoonotic parasite given opportunity due to permissive ecological circumstances [85–88].

Phylogenetic and historical isolation of giant pandas from the broader assemblage of ursids and ursine bears (e.g. [23, 24]) in conjunction with apparent structural divergence (e.g. teeth and configuration of the dorsal ray; Table 2 and Fig. 5b5 and 10) of *A. ailuropodae* and *A. malayanum* suggests that independent events of host colonization, separated in space and time, were essential in the process of speciation for these hookworms; molecular data, particularly from *A. malayanum*, is still needed to explore this hypothesis. Moreover, phylogenetic hypotheses for the Ursidae have placed giant pandas distantly from species of *Ursus* (and other ursines) near the base of an extensive radiation for bears that unfolded across the late Miocene and Pliocene [24]*.* Among ursine hosts for *Ancylostoma*, *U. thibetanus* (Asiatic black bear) is regarded as the sister of *U. americanus* (American black bear) and placed among crown species in ursid phylogenies [23, 24]. These relationships alone would serve to refute a coevolutionary hypothesis for *Ancylostoma* hookworms among bears, conversely supporting a history of independent events of host colonization that have structured this fauna.

Unlike *U. yukonensis* and *U. rauschi* in bears, the hookworm from giant panda is genetically similar to other *Ancylostoma* species (Fig. 7). These respective genera are referred to two independent subfamilies within the Ancylostomatide, namely Ancylostomatinae Looss, 1905 for *Ancylostoma* and Bunostominae Looss, 1911 for *Uncinaria*, consistent with extended evolutionary trajectories for these taxa among the hookworms. This suggests the independent origin of *A. ailuropodae*, supporting monophyly of *A. ailuropodae* and congeneric species *A. ceylanicum*, *A. duodenale*, *A. tubaeforme*, *A. caninum* and *A. braziliense*, and strengthens the close relationship between the giant panda hookworm and *A. ceylanicum* within the clade. Concurrently it suggests that *Uncinaria* spp. from pinnipeds and ursids are a distinct monophyletic group in the family Ancylostomatidae [70]. The apparent genetic differences of *A. ailuropodae* n. sp. in pandas and *U. rauschi* and *U. yukonensis* in bears, coupled with their divergent biogeographic and ecological histories suggest this system as a good model for exploring the complexities of diversification and faunal assembly in the evolution of host range and associations among hookworms (e.g. [85–88]).

The potential for genetic partitioning among possible disjunct populations of hookworms in giant pandas should be considered, as it will reflect information about the timing of colonization to giant pandas and the duration of the history of association. Further, the history of fragmentation and isolation for giant pandas across now isolated mountain systems in southwestern China suggests a complex relationship among hosts and hookworms in this region. Such history could be explored through fecal-based approaches in conjunction with molecular diagnostics to examine occurrence and the extent of genetic diversity and distribution for hookworm parasites among populations and subspecies of giant pandas.

## Conclusions

This study is the first to describe and define a new member of the genus *Ancylostoma*, *A. ailuropodae*, in the wild giant panda using morphological and molecular criteria. Morphological characters (e.g. ventrolateral (two pairs) and dorsolateral (two pairs) teeth and dorsal rays) distinctly separate *A. ailuropodae* n. sp. from other congeneric species in the genus *Ancylostoma*. Further, nuclear ITS1-5.8S-ITS2 and mitochondrial *cox*1-based genetic distance analysis and phylogenies supported the assertion that *A. ailuropodae* is independent and shares a sister-species relationship with the anthropozoonotic *A. ceylanicum*. Although additional molecular evidence is warranted, this finding should enhance public awareness of parasitic hookworms in giant pandas, especially in captive populations that have frequent contact with breeders, veterinarians and even tourists. Moreover, the morphological and molecular data presented here enhances the information on species within the genera *Ancylostoma*, *Uncinaria*, and *Necator* and contributes to a more complete understanding of the taxonomy, diagnostics and evolutionary biology of hookworms.

## Abbreviations

BF: Bootstrapping frequencies
BI: Bayesian inference
BIC: Bayesian information criteria
*cox*1: Cytochrome *c* oxidase subunit 1
ITS: Internal transcribed spacer (ITS1-5.8S-ITS2)
MCMC: Markov chain Monte Carlo
MP: Maximum parsimony
MYA: Million years ago
PP: Posterior probabilities
SD: Standard deviation
TBR: Tree-bisection-reconnection
